# Weight bearing versus conventional CT for the measurement of patellar alignment and stability in patients after surgical treatment for patellar recurrent dislocation

**DOI:** 10.1007/s11547-021-01339-7

**Published:** 2021-03-03

**Authors:** Giada Lullini, Claudio Belvedere, Maurizio Busacca, Antonio Moio, Alberto Leardini, Silvio Caravelli, Bruna Maccaferri, Stefano Durante, Stefano Zaffagnini, Giulio Maria Marcheggiani Muccioli

**Affiliations:** 1 II Clinica Ortopedica E Traumatologica, IRCCS Istituto Ortopedico Rizzoli, Bologna, Italy; 2 Movement Analysis Laboratory, IRCCS Istituto Ortopedico Rizzoli, Bologna, Italy; 3 Radiologia Diagnostica Ed Interventistica, IRCCS Istituto Ortopedico Rizzoli, Bologna, Italy; 4 DIBINEM - University of Bologna, via di Barbiano, Bologna, Italy; 5 Nursing Technical and Reahabilitation Assistance Service, IRCCS Istituto Ortopedico Rizzoli, Bologna, Italy

**Keywords:** Weight-bearing, Cone-beam CT, Conventional CT, Patellofemoral instability

## Abstract

**Purpose:**

To compare weight-bearing cone-beam computer tomography (CBCT) and conventional computer tomography (CT)-based measurements of patellofemoral alignment and stability in patients surgically treated for recurrent patellar dislocation. These scans implied respectively single-leg up-right posture, the knee flexed, and lower limb muscles activation, versus supine position with the knee extended.

**Methods:**

A total of 17 patients (11 males/6 females) after surgical reconstruction with fascia lata allograft for recurrent patellofemoral dislocation were analyzed at 60-month follow-up. Tilt and congruence angles and tibial tuberosity–trochlear groove (TT-TG) offset were measured on images obtained from CBCT and conventional CT scans by three independent and expert radiologists. Paired t tests were performed to compare measurements obtained from the two scans. Inter-rater reliability was assessed using a two-way mixed-effects model intra-class correlation coefficient (ICC).

**Results:**

Only TT-TG offset was found significantly smaller (*p* < 0.001) in CBCT (mean 9.9 ± 5.3 mm) than in conventional CT (mean 15.9 ± 4.9 mm) scans. ICC for tilt and congruence angles and for TT-TG offset ranged between 0.80–0.94 with measurements in CBCT scans, between 0.52 and0.78 in conventional CT.

**Conclusion:**

In patients surgically treated for recurrent patellar dislocation, TT-TG offset was found overestimated with conventional CT. All measurements of patellofemoral stability and alignment were found more consistent when obtained with weight-bearing CBCT compared to conventional CT.

## Introduction

The occurrence of patellar instability is a common event in orthopedics, where chronic patellar dislocation is estimated to affect 5.8 cases every 100,000 people, becoming even five times larger in the young population, aged between 10 and 17 years [1, 2]. Patello-femoral joint stability is guaranteed by the combination of a complex and multifactorial equilibrium. Patellar tracking is indeed influenced by knee joint bone morphology, ligaments integrity, and lower limb muscles activation, especially of quadriceps [3]. Different surgical options are available for the treatment of recurrent patellar dislocation either individually or in combination: medial patellofemoral ligament (MPFL) reconstruction, tibial tubercle distalization, trochleoplasty, and tibial or femoral osteotomy for correction of rotational or coronal plane malalignments [4]. Recent studies have shown that anatomical MPFL reconstruction is the gold standard technique to treat recurrent patellar luxation/instability, this being executed in isolation or in combination with other surgical procedures [5].

The choice for single or combined techniques for this surgical treatment needs to be assessed according to patient-specific patho-anatomy. Therefore, medical imaging is fundamental to assess factors predisposing to instability and to determine the best treatment planning for each specific patient [6]. Nowadays, imaging of the patellofemoral joint is performed with conventional radiographs, magnetic resonance imaging (MRI), and conventional computed tomography (CT) scans [7].

Medical images are essential to measure patellofemoral stability and alignment parameters, in particular the patellar tilt angle, congruence angle, and the distance between the tibial tubercle and the center of the trochlear groove, i.e., the TT-TG offset [7]. Conventional CT scans are performed with patient in supine position, with the knee fully extended and the lower limb muscles relaxed, thus far from the physiological weight-bearing conditions. It is known that quadriceps muscular contraction and knee flexion which occur in weight-bearing influence patellar stability and alignment [8]. Moreover, the evidence from the literature supports the fact that removing these conditions can affect stability and alignment parameters measurement [9–12].

The cone-beam CT technology (CBCT) has recently allowed new devices to collect scans at lower limbs in realistic and physiological weight-bearing conditions. The original raw data-set is three-dimensional (3D), but standard bi-dimensional views in Dicom format are available in a few minutes [13, 14]. In a few previous studies, CBCT scanners have been used already to investigate variations of patellofemoral stability and alignment parameters between the standard supine position obtained with conventional CT and the weight-bearing position from CBCT, in healthy subjects [15, 16] and in patients diagnosed with lateral patellar instability [17]. To the best of the authors’ knowledge, no studies have been conducted yet in patients surgically treated via MPFL for recurrent patellar instability.

The purpose of the present study was to compare patellofemoral stability and alignment parameters measured with CBCT under weight-bearing and with knee flexion of 30°, with those obtained with conventional CT in supine position in patients treated with MPFL reconstruction for recurrent patellar dislocation, at medium-term follow-up. The hypothesis was that measurements of patellofemoral stability and alignment parameters acquired with weight-bearing CBCT scans would be different from those obtained with conventional CT.

## Material and methods

### Patient population

A total of 19 patients treated at our Institution between 2012 and 2013 with minimally invasive arthroscopically assisted MPFL reconstruction using fascia lata allograft for recurrent chronic patellar dislocation were enrolled in this study. A total of 17 patients were available at 60-month follow-up for conventional CT and weight-bearing CBCT scans at author’s institution. The inclusion criteria for surgery were: (1) positive history for at least two episodes of patellar dislocation, or (2) a single episode followed by a permanent feeling of patellar instability during daily life activities, non-respondent to 6 months of physical therapy, (3) traumatic etiology (first episode), and (4) MPFL lesion confirmed by a positive glide test or apprehension test. The exclusion criteria were: (1) previous surgical bony procedure on the affected or contralateral knee; (2) age < 15 years; and (3) presence of a congenital hyper-laxity syndrome (Marfan, Down’s syndromes, etc.). The study protocol was approved by the local ethical committee. All patients gave informed consent to surgery and to enrollment in the present study according to European Union laws, in accordance with the ethical standards of the institutional and/or national research committee and with the 1964 Helsinki Declaration and its later amendments or comparable ethical standards. Complete preoperative patients’ demographic data are reported in Table 1.

**Table 1:** Demographic data

Gender (Male/Female)	11 / 6
Age at surgery (years)	21.7 ± 5.1 (min ÷ max: 15.9 ÷ 30.3)
BMI	24 ± 3.7 kg/m2
Mean follow-up (months)	64.3 ± 12.5
Affected side (Right/Left)	9/8
Previous procedures	3 lateral releases 1 multiple soft tissue tensioning

The employed MPFL reconstruction technique was already published [18] and did not show in vitro adverse biomechanical effects (e.g., patellar mal-tracking or higher articular contact pressure) [19]. Patients underwent MPFL reconstruction either as an isolate procedure or in combination with other patellar stabilizing surgeries (Table 2). All surgical interventions were performed by the same senior author. In case of patella alta, defined as a Caton–Deschamps index [20] larger than 1.4, a tibial tuberosity distalization was also performed. In the case of TT-TG distance larger than 20 mm, a medialization of the tibial tuberosity was also performed. If trochlear dysplasia was diagnosed, as described by Dejour [21], the decision to perform a type D trochleoplasty was taken intra-operatively. After surgery, a brace in full extension was prescribed for 4 weeks; afterward, patients followed a standard 5-month rehabilitation program for patellofemoral instability [22].

**Table 2:** Concurrent surgical procedures

Surgical treatment	Frequency	%
Isolated MPFL reconstruction	9	53%
MPFL reconstruction + TT distalization	1	6%
MPFL reconstruction + TT distalization/medialization	5	29%
MPFL reconstruction + TT dist/med + trochleoplasty	2	12%

MPFL, medial patello-femoral ligament; TT dist/med, tibial tuberosity diastalization/medialization

### Data collection

**Fig. 1:**
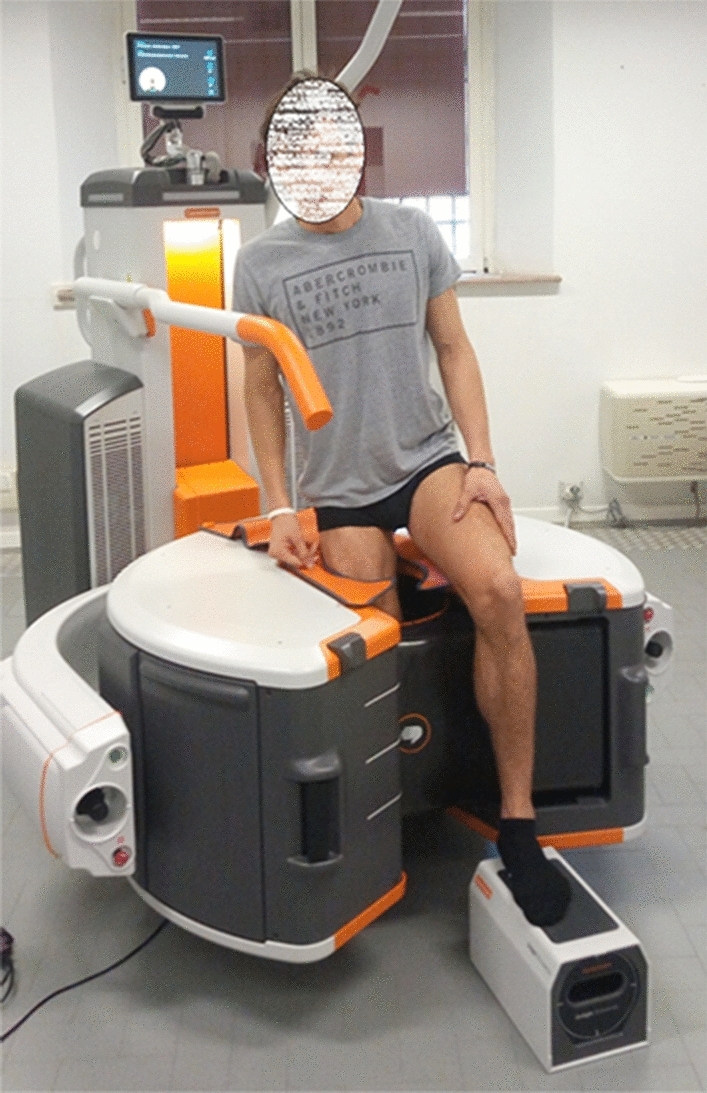
Picture taken from one typical patient during scan with the CBCT in weight-bearing

**Fig. 2:**
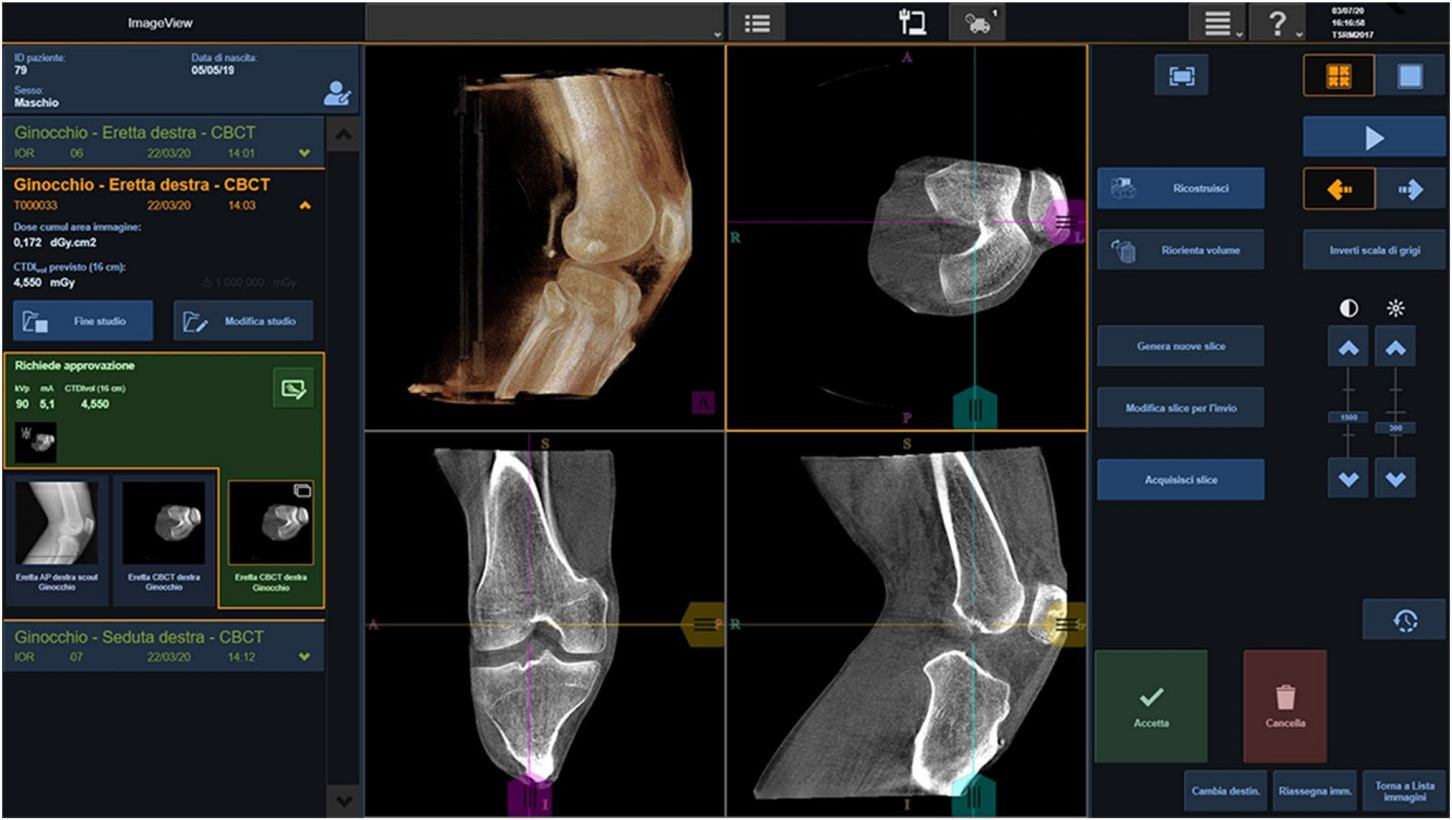
Screenshot from CBCT device with of the three anatomical plus the 3D rendering views

**Fig. 3:**
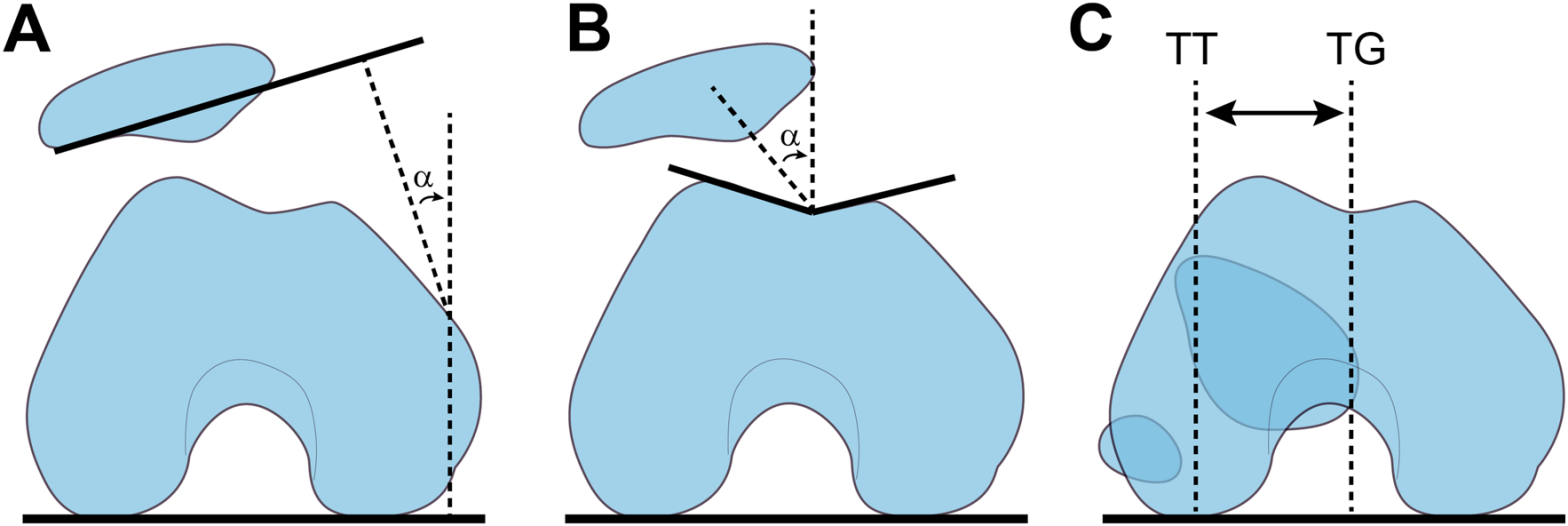
Schematic drawings of the methods used to measure** a** tilt angle,** b** congruence angle, and** c** tibial tuberosity–trochlear groove (TT-TG) offset distance on axial two-dimensional computed tomography images

At 60-month follow-up, all treated patients were contacted for follow-up. In detail, available patients underwent a conventional CT scan performed in supine position, with knee in full extension and leg muscles relaxed, as part of standard care to assess results at mid-term follow-up. This was performed on General Electric CT ‘Revolution Discovery GSI/HD CT’ (GE Healthcare, Chicago, IL-USA): acquisition helical full 1 s; high resolution; DFOV 180 mm; slice thickness 0.625 mm; interslice 0.312 mm; matrix 512 mm; kV 120–140; mA.s 130–146; CT dose index vol 13.29 mGy; dose-length product 153.9–267.3 mGy cm. On the same day, patients underwent also a CBCT scan (Fig. 1), with patient in single-leg up-right posture, in full weight-bearing on the operated limb, with the knee flexed at 30° thus with leg muscles active (Fig. 2). This was performed on ‘OnSight 3D Extremity System’ (Carestream, Rochester, NY-USA): slice thickness 0.26 × 0.26 mm; radiation exposure (vol) 4.55 mGy; dose-length product 172 mGy cm; 5.1 mA; 90 kV. Knee position and leg load were checked, respectively, with a goniometer and a plantar pressure pad. Relevant images from both CT scans were analyzed by three independent radiologists with large experience in musculoskeletal images. The tilt angle, the congruence angle, and the TT-TG offset were measured with the digital image tools available in a PACS viewing workstation, and as described in the literature (Fig. 3). [17, 21, 23, 24]

### Statistical analysis

Descriptive statistics were calculated for patellofemoral measurements. Paired *t* test stratified by rater were used to compare these patellofemoral measurements obtained from conventional CT versus CBCT scans. One-way analysis of variance was used to test the difference in terms of means among raters.

Inter-rater reliability was assessed using a two-way mixed-effects model intra-class correlation coefficient (ICC) with corresponding 95% CI for tilt angle, congruence angle, and TT-TG offset on conventional and CBCT scans. ICC values smaller than 0.50, between 0.50 and 0.75, between 0.75 and 0.90, and greater than 0.90 are indicative of poor, moderate, good, and excellent reliability, respectively [25]. A priori power analysis was performed to define the sample size. A *p* value of less than 0.05 was considered to be statistically significant; otherwise, the nonsignificance was indicated. The size of the patient sample analyzed in the present study was estimated by considering previous relevant literature [16, 17], reporting the same acquisition technique as in the present study, and meets the criteria for achieving differences in measurements with 80% statistical power and a level of 0.05.

## Results

**Fig. 4:**
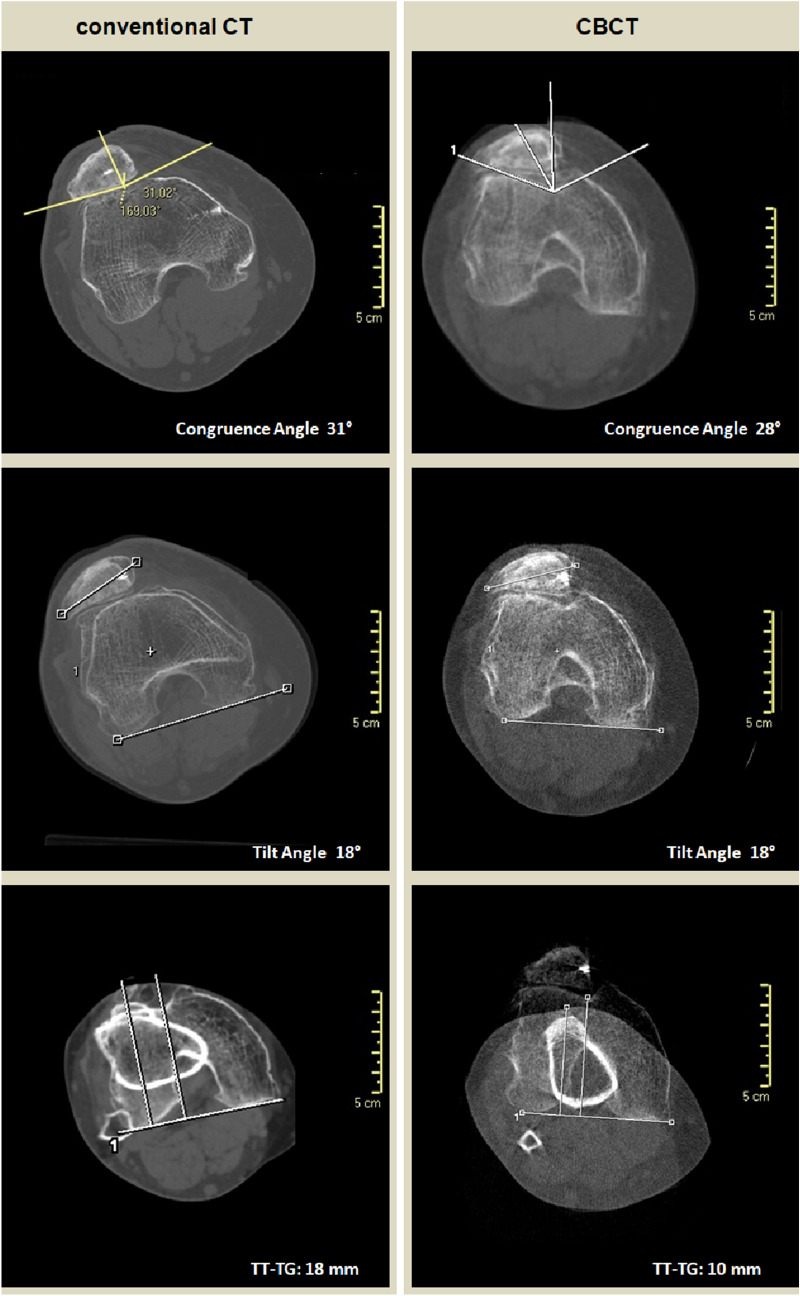
Measurements of patellofemoral stability and alignment on conventional computer tomography (CT) images and weight-bearing cone-beam CT (CBCT) performed on a single patient

The TT-TG offset was significantly less when acquired with the CBCT scan (mean 9.9 mm ± 5.3 mm) compared to the conventional CT scan (mean 15.9 mm ± 4.9 mm) (*P* < 0.001) for all the three raters. Table 3 shows a comparison between measurements of patellofemoral stability and alignment on conventional CT images versus CBCT images, stratified by rater and by the average for the three raters. Differences between mean values for each measurement of patellofemoral stability and alignment for the three raters are presented in Table 4. Inter-rater reliability for tilt angle, congruence angle, and TT-TG offset was good if measured with CBCT scan (ICC range: 0.80–0.94), average if measured with conventional CT (ICC range: 0.52–0.78). Results are presented in Table 5. Intra-rater reliability was not assessed. Figure 4 shows the measurements of patellofemoral stability and alignment on conventional CT images and CBCT images performed on a single patient.

**Table 3:** Comparison of Conventional CT Versus CBCT Patellar Measurements by Rater

	Rater 1			Rater 2			Rater 3			Average for 3 Raters		
	Conv. CT	CBCT	*p* valu*e*	Conv. CT	CBCT	*p*	Conv. CT	CBCT	*p*	Conv. CT	CBCT	*p* value
Congruence Angle (deg)	31.9 ± 13.0	34.1 ± 20.0	0.62	17.2 ± 13.1	24.6 ± 13.2	0.03	20.4 ± 16.8	24.2 ± 19.1	0.50	23.1 ± 15.5	27.7 ± 17.9	0.08
Tilt Angle (deg)	15.6 ± 13.2	16.8 ± 10.8	0.54	16.2 ± 8.0	14.1 ± 9.3	0.41	15.4 ± 9.0	12.7 ± 10.3	0.17	15.7 ± 10.1	14.5 ± 10.1	0.32
TT-TG (mm)	16.5 ± 4.2	9.4 ± 5.1	< .001	15.9 ± 5.7	10.6 ± 5.4	< .001	15.2 ± 4.8	9.8 ± 5.5	< .001	15.9 ± 4.9	9.9 ± 5.3	< .001

Mean ± SD of data derived from conventional (conv. CT) and cone-beam (CBCT) computed tomography, along with relevant p value as by paired t test, for each of the three raters

**Table 4:** Comparison of conventional CT versus CBCT patellar measurements between raters by type of CT

	conventional CT				CBCT			
	Rater 1	Rater 2	Rater 3	*p* valu*e*	Rater 1	Rater 2	Rater 3	*p* valu*e*
Congruence angle (deg)	31.9 ± 13.0	17.2 ± 13.1	20.4 ± 16.8	0.01	34.1 ± 20.0	24.6 ± 13.2	24.2 ± 19.1	0.19
Tilt Angle (deg)	15.6 ± 13.2	16.2 ± 8.0	15.4 ± 9.0	0.96	16.8 ± 10.8	14.1 ± 9.3	12.7 ± 10.3	0.50
TT-TG (mm)	16.5 ± 4.2	15.9 ± 5.7	15.2 ± 4.8	0.73	9.4 ± 5.1	10.6 ± 5.4	9.8 ± 5.5	0.80

Data are presented as mean ± SD. CT, computed tomography; CBCT, cone beam computed tomography; conv. CT, conventional computed tomography; Cong. Angle, congruence angle; TT-TG, tibial tuberosity–trochlear groove

**Table 5:** Inter-rater reliability by type of CT

	Conventional CT		CBCT	
	ICC	*p* value	ICC	*p* value
Congruence Angle (deg)	0.73	< 0.001	0.80	< 0.001
Tilt Angle (deg)	0.52	0.04	0.80	< 0.001
TT-TG off-set (mm)	0.78	< 0.001	0.94	< 0.001

Intraclass correlation coefficient (ICC) for the three parameters as derived from conventional (conv. CT) and cone-beam (CBCT) computed tomography

## Discussion

The most important finding of the present study was that measurements of TT-TG offset, in patients after surgical treatment for recurrent patellar dislocation, was reduced on images obtained from weight-bearing CBCT compared to those obtained with conventional CT. Moreover, all measures of patellofemoral stability and alignment were more consistent when obtained with CBCT compared to conventional CT.

Conventional CT is still widely used to assess patellofemoral stability and alignment, but images executed with the patient supine and the knee fully extended do not reflect real in-vivo conditions and may affect measurements.

In the literature, there is controversy on whether magnetic resonance imaging and CT-based measurements of patellar tilt, subluxation, and, particularly, of TT-TG offset may be equivalent [7]. However, some image-based studies conducted via MRI with patients in weight-bearing and/or with the knee flexed, opened the way to further investigations of patellofemoral stability and alignment in these conditions. By using MRI and with patient supine, it has been shown that there is a significant progressive reduction in TT-TG offset when the knee is imaged from 0° to 90° [9–12]. Real-time MRI was used to acquire patellofemoral kinematics during dynamic knee extension from 30° to 0° in supine mimicking weight-bearing [26]; this study advocates that patellofemoral kinematics measured during supine position does not accurately represent patellar motion during weight-bearing activities. A more recent MRI study [10] investigated the effect of weight-bearing on TT-TG offset when measured with patients in real weight-bearing condition. In this study, the mean TT-TG offset was significantly less at both 0° and 30° of knee flexion compared to the lying position with the knee at 0° of flexion.

The modern CBCT scanners seem capable to collect knee scans finally with patients in weight-bearing position and with the knee joint flexed, potentially enabling to get access to measurements for patellofemoral stability and alignment in more physiological conditions. In a previous study by Hirschmann et al. [15], the effects of weight-bearing on joint alignment was investigated in healthy subjects. It was concluded that knee joint alignment change significantly in the upright weight-bearing position compared to supine. In a recent study by Marzo et al. [17], measurements of patellofemoral stability and alignment obtained from conventional CT were compared to those of CBCT in patients suffering from patellofemoral instability. Tilt and congruence angles and TT-TG offset were all found significantly smaller under weight-bearing condition compared to conventional CT scan. Later, the same authors investigated the possible TT-TG offset variation between conventional CT and CBCT in a population of normal subjects [16] and concluded that also in this case CBCT scans result in significantly smaller values.

The present study is the first comparing postoperative patellofemoral stability and alignment parameters measured from CBCT scans, with patients in weight-bearing and the knee flexed, and those measured from conventional CT scans, in supine position and knee extension.

To assess patellofemoral stability and alignment, the tilt angle, the congruence angle, and the TT-TG offset were chosen as imaging parameters. These measurements are considered among essential patellofemoral stability and alignment criterion and are the most commonly presented in the literature [7, 16, 17, 21]. Moreover, previous studies have documented an excellent inter- and intra-rater reliability for these parameters except for the congruence angle that demonstrated a lower reproducibility. [27] Normality values of these measures are reported in the literature [21, 23].

In the present study, the TT-TG offset was found significantly smaller in images obtained from the CBCT versus the conventional CT scanner. This result is in accordance with what reported previously by Marzo et al. [16] and Hirschmann et al. [15] in different populations. It is known that changes in the TT-TG offset occurring along knee flexion are most likely a result of the unlocking of the screw-home mechanism of the tibiofemoral joint, which occurs in early flexion and which results also in internal rotation of the tibia with respect to the femur, and therefore of the tibial tuberosity with respect to the femoral trochlea [10, 12].

The presented measurements of the tilt and congruence angles were influenced by the exact knee flexion angle and the muscle activation experienced in the single-leg up-right postures. The tilt angle was found smaller, even though statistically not significant, which concurs with results from Marzo et al. [17] For the congruence angle, an opposite variation with respect to Marzo et al. [17] was found, though not significant: These three independent raters in fact measured values for this parameter larger in CBCT than in conventional CT scans. In a recent meta-analysis [27], this parameter demonstrated, however, a poor discrimination validity and a high statistical heterogeneity, which can certainly justify this result. The present assessment of inter-rater reliability between the three radiologists (Tables 4 and 5) points out that this was good in CBCT measurements (ICC range 0.80–0.94) but average in conventional CT. This suggests that all measures of patellofemoral stability and alignment were more consistent when obtained with CBCT compared to conventional CT.

Despite the fact that no standards exist in patients positioning during MRI- or CT-based image acquisitions, it is here confirmed that overall posture and knee loading condition affect considerably the patellofemoral stability and alignment related measurements. It is straightforward, in the clinical practice, that these and other biomechanical measures influence much indication and surgical strategy for all patients with chronic patellar instability. As shown, a conventional CT scan performed in full knee extension and in no weight-bearing may overestimate the TT-TG offset and provide misleading information to surgeons considering realignment surgery. In particular, this overestimation of the degree of patellar malalignment before surgery can lead to a corresponding overcorrection in surgery, with subsequent likely complications such as knee pain for abnormal joint loading and arthritis.

The present study has a number of limitations.

First, the number of patients involved is limited, mainly because of the low incidence in the general population of the pathology here investigated. Secondly, the present patient population is also not homogeneous concerning the surgical treatment, because MPFL reconstruction was frequently accompanied with other treatments. This aspect, however, is frequent in studies on patellofemoral surgery because chronic joint instability is a multi-factorial disease that must be addressed with à-la carte surgical intervention. Moreover, the addition of a control group made of by the same subjects in the preoperative assessment could be useful to better understand how the difference between CBCT and conventional-CT would affect surgical decision making. Unfortunately this study was already performed by Marzo et al. [17] and ethical limitations related to double-radiation exposure was not accepted by our local ethic committee. In addition, a control group of healthy subjects for physiological corresponding measurements would have been valuable, but again ethical limitations related to radiation exposure was not accepted by our local ethic committee.

Furthermore, everyday use of CBCT in preoperative study and postoperative control is restricted due to the limited availability of CBCT equipment to perform weight-bearing examinations.

All these limitations, however, does not weaken the original main scope of the study, which is a comparison between CT scan conditions in the postoperative state of these patients. This suggests, as found also by Marzo et al. [17] in the preoperative condition, that measurements of TT-TG offset are smaller in weight-bearing CBCT; the real values in this condition could be a better parameter to give the indication for TT-TG transposition.

## Conclusion

In patients surgically treated with MPFL reconstruction with fascia lata allograft for recurrent patellar dislocation, measurements of TT-TG offset are reduced on images obtained from weight-bearing CBCT scans with flexed knee compared to those obtained from conventional CT with patient supine and extended knee. Moreover, all analyzed measurements of patellofemoral stability and alignment obtained with CBCT were more consistent than those of conventional CT.

## Declarations

### Human and animal participants

The research involved human participants. They signed an informed consent before enrolment into the study.
